# The Ground-Based BIOMEX Experiment Verification Tests for Life Detection on Mars

**DOI:** 10.3390/life11111212

**Published:** 2021-11-09

**Authors:** Claudia Pacelli, Alessia Cassaro, Ilaria Catanzaro, Mickael Baqué, Alessandro Maturilli, Ute Böttger, Elke Rabbow, Jean-Pierre Paul de Vera, Silvano Onofri

**Affiliations:** 1 Italian Space Agency, Via del Politecnico snc, 00133 Rome, Italy; claudia.pacelli@asi.it; 2 Department of Ecological and Biological Sciences, University of Tuscia, Largo dell’Università snc, 01100 Viterbo, Italy; ilaria.catanzaro@unitus.it (I.C.); onofri@unitus.it (S.O.); 3 Research Group Astrobiological Laboratories, Institute of Planetary Research, Management and Infrastructure, German Aerospace Center (DLR), Rutherfordstraße 2, 12489 Berlin, Germany; Mickael.Baque@dlr.de (M.B.); alessandro.maturilli@dlr.de (A.M.); 4 Institute of Optical Sensor Systems, German Aerospace Center (DLR), Rutherfordstraße 2, 12489 Berlin, Germany; Ute.Boettger@dlr.de; 5 Institute of Aerospace Medicine, Radiation Biology, German Aerospace Center (DLR), Linder Höhe, 51147 Cologne, Germany; elke.rabbow@dlr.de; 6 Space Operations and Astronaut Training, MUSC, German Aerospace Center (DLR), Linder Höhe, 51147 Cologne, Germany; jean-pierre.devera@dlr.de

**Keywords:** biosignature, dark pigments, polysaccharides, polychromatic radiation, simulated conditions, Mars, life-detection

## Abstract

The success of an astrobiological search for life campaign on Mars, or other planetary bodies in the Solar System, relies on the detectability of past or present microbial life traces, namely, biosignatures. Spectroscopic methods require little or no sample preparation, can be repeated almost endlessly, and can be performed in contact or even remotely. Such methods are therefore ideally suited to use for the detection of biosignatures, which can be confirmed with supporting instrumentation. Here, we discuss the use of Raman and Fourier Transform Infrared (FT-IR) spectroscopies for the detection and characterization of biosignatures from colonies of the fungus Cryomyces antarcticus, grown on Martian analogues and exposed to increasing doses of UV irradiation under dried conditions. The results report significant UV-induced DNA damage, but the non-exceeding of thresholds for allowing DNA amplification and detection, while the spectral properties of the fungal melanin remained unaltered, and pigment detection and identification was achieved via complementary analytical techniques. Finally, this work found that fungal cell wall compounds, likely chitin, were not degraded, and were still detectable even after high UV irradiation doses. The implications for the preservation and detection of biosignatures in extraterrestrial environments are discussed.

## 1. Introduction

The detection of biosignatures on Mars is of outstanding interest in the current field of astrobiology, and drives various fields of research, ranging from new sample collection strategies to the development of more sensitive detection techniques. The effects of radiation on the stability of biosignatures are essential. On Earth, the preservation of organic biosignatures is principally influenced by the rates of biological records production and alterations from biological recycling and abiotic factors (e.g., radiation), the latter of which must not exceed the former. Some types of biosignatures have been found to be more resistant to certain degradation processes, while some environments may be more favorable in preserving some types than others [1,2,3]. Research on these issues is a driving factor in designing explorative strategies for life-detection missions throughout the Solar System.

Ultraviolet (UV) radiation, especially shorter-wavelength UVC radiation, is known to be a damaging factor for organisms and potential organic biosignatures. On Mars, for example, exposure to the surface UV radiation can degrade common biologically relevant organic molecules to simple organics that may confound identification via biosignatures, even on relatively recently exposed surfaces [4,5,6,7]. On Earth, the interiors of rocks provide a protected environment for extant life in some of the most inhospitable terrestrial environments, including the Antarctic Dry Valleys. The endolithic habitat provides thermal buffering, physical stability, and protection from incident UV, solar radiation, and dryness [8]. Microorganisms can be found at depths of a few to tens of millimeters in rock interiors; these are so-called cryptoendolithic organisms.

The UV environment on Mars differs significantly from that on present-day Earth, and it can significantly influence how biosignatures persist. The Martian atmosphere (primarily composed of CO_2_) effectively absorbs all UV radiation below 190 nm and has a modest absorbing effect from 190 to 200 nm, but atmospheric absorbance at longer wavelengths is negligible, and therefore UV with wavelengths >200 nm interacts with the surface [9]. On Earth, most of the UVB (280–315 nm) and UVC (200–280 nm) radiation is absorbed by the ozone (O_3_); conversely, this molecule is present in low concentrations in the current Martian atmosphere [10]. The actual UV flux reaching the surface of Mars is influenced by various factors, such as latitude, season, and the orbital position of the planet, and can also fluctuate due to small seasonal build-ups of O_3_, as well as dust load in the atmosphere [9,11]. The average UV irradiance (200–400 nm) at the Martian surface has been modeled to be ~50 J/(s⋅m^2^) [12,13]. UVB and UVC represent Mars’ UV incident radiation wavelengths that are extremely harmful to DNA and other common biological molecules [4,5,7,14]. Several direct measurements of UV irradiance have been made by the Curiosity rover, showing that the maximum total UV flux at the Gale Crater may be closer to 20 J/(s⋅m^2^), which is less severe than the typically modeled irradiance level but is still not favorable for surface habitability [11]. Additional factors, such as ionizing radiation, the presence of oxidants or other reactive species in the atmosphere, and regolith on Mars, would appear to make the long-term survival of organisms or organic biosignatures on the surface highly unlikely [15,16,17,18,19,20].

Considering that few works have dealt with the interactions of incident UV flux with minerals and rocks [9,21,22,23], even in preparation for in situ missions to Mars (such as ExoMars and Mars 2020 [24,25]), the issue is not yet well understood, and therefore a further necessary step is to identify the extent to which rocks and minerals can provide effective shielding against UV radiation.

The present study was performed in the context of the Biology and Mars Experiment (BIOMEX), part of the EXPOSE-R2 mission of the European Space Agency (ESA) along with three other astrobiological experiments, which facilitated 16-month exposure on the outer side of the International Space Station (ISS) from August 2014 to February 2016 [26,27]. The aim of the study was to investigate the resistance and stability of biomolecules to UV irradiation, assessed via the Experiments Verification Test (EVT) ground-based experiments in preparation for the space-exposure BIOMEX project, as support for the ongoing life-detection missions on Mars (e.g., ExoMars’ Rosalind Franklin mission). These included the exposure of *C. antarcticus* MNA-CCFEE 515, a cryptoendolithic black fungus isolated from Antarctic rocks, to increasing doses of polychromatic UV (200–400 nm) irradiation (up to 5.5 × 10^5^ kJ/m^2^). Samples were grown on Martian mineral analogues (Phyllosilicatic and Sulfatic Mars Regolith Simulants) and dried before the irradiation treatment. The stability of fungal biomolecules within Martian analogue minerals was investigated through quantitative PCR (qPCR), UV–Visible (UV-Vis) spectrophotometry, and Raman and FT-IR spectroscopies.

## 2. Materials and Methods

### 2.1. Sample Preparation and Cultivation Conditions

The cryptoendolithic black fungus *C. antarcticus* MNA-CCFEE 515 of the Mycological Section of the University of Tuscia (Viterbo, Italy) was isolated by R. Ocampo-Friedmann from sandstone collected by H. Vishniac at Linnaeus Terrace in McMurdo Dry Valleys (Southern Victoria Land, AQ) during the 1980-1981 Antarctic expedition [28].

The fungal cell suspension was spread on Malt Extract Agar (MEA) medium (malt extract, powdered: 30 g/L; peptone: 5 g/L; agar: 15 g/L; Applichem GmbH) in Petri dishes and mixed with three separate substrates: Antarctic sandstone (15 g/L) and two Martian regolith analogues (10 g/L). Antarctic sandstone was the original substrate (OS) where the fungus occurs naturally, while the Phyllosilicatic Mars Regolith Simulant (P-MRS) and the Sulfatic Mars Regolith Simulant (S-MRS) mimicked the regolith of the phyllosilicate deposits formed mainly on early Mars and the regolith of sulfate deposits currently observed on Mars, respectively. The mineralogical composition of both Martian analogues was provided by Böttger et al. [29]. These Martian regolith analogues have been chosen to evaluate whether they may interfere with fungal biomolecule detection.

All samples were incubated for 3 months at 15 °C; once fungal colonies were grown, circular portions were cut to fit within the wells (12 mm) of the exposure carrier and then dehydrated in a sterile hood at room temperature overnight.

### 2.2. Test Facilities and Exposure Conditions

In the context of the BIOMEX project, ground-based environmental and space simulations were performed using the Planetary and Space Simulation facilities (PSI) at the Institute of Aerospace Medicine (German Aerospace Center, DLR, Köln, Germany). The exposure conditions of the two parts of the EVTs (Experiment Verification Test part 1: performed to simulate several space and Mars-like parameters; Experiment Verification Test part 2: performed to simulate the UV irradiance as expected during a 12-month mission in Low Earth Orbit) were reported by de Vera et al. [26].

All tests were performed in triplicate, and untreated samples, kept in the dark at room temperature, were used as laboratory controls (CTRs). Samples of each substrate were either not irradiated or irradiated with one of the fluences from 5.5 × 10^2^ km^2^ up to 5.5 × 10^5^ kJ/m^2^ in the respective irradiation times using a Solar Simulator at 1271 J/s·m^2^ as listed in Table 1. For each substrate (OS, P-MRS, S-MRS), we investigated pivotal samples from the EVT2 treatment (i.e., CTRs, 5.5 × 10^2^ kJ/m^2^, and 5.5 × 10^5^ kJ/m^2^ samples; Table 1); moreover, the *C. antarcticus* colonies grown under physiological conditions on MEA without substrate mixing were used as positive controls (POS CTRs).

### 2.3. DNA Integrity Assay

#### 2.3.1. DNA Extraction and Quantification

Genomic DNA was extracted from the selected EVT2 and POS CTR samples using NucleoSpin Plant II Kit/2011 (MACHEREY-NAGEL GmbH & Co. KG, Düren, Germany) and following the protocol optimized for fungi [28].

Samples were quantified using the Qubit Fluorometer (Invitrogen) and the Qubit dsDNA High Sensitivity (HS) Assay Kit (Invitrogen). The assay was performed at room temperature, and three readings for each sample were performed. Dilution factors were calculated after achieving sample concentrations, and DNA samples were diluted up to the same concentration (0.018 ng/μL) before running the qPCR analysis.

#### 2.3.2. DNA Amplification

A real-time qPCR was conducted to quantify the number of fungal Large Subunit (LSU) ribosomal DNA restricted fragments (~330 bp) using LR3R (GTC TTG AAA CAC GGA CC) [31] and LR5 (TCC TGA GGG AAA CTT CG) [32] primers. The qPCR reactions were performed in the Bio Rad CFX96 Real-Time PCR Detection System, and the Bio Rad CFX Manager software processed the results in graphs. The reactions were carried out in triplicate in the 96-well plate with a solution containing 7.5 μL iQ SYBR Green Supermix (Bio Rad, Milan, Italy), 1.0 μL each primer solution (5 pmol/µL), and 5.5 ng DNA template in a final volume of 15 μL; for the No Template Control (NTC) sample, 5.5 μL sterile deionized water was added instead of DNA template.

The amplification conditions of the LSU region were derived from Selbmann et al. [33]. Fluorescence measurements were performed at the end of each annealing cycle. A melt curve analysis was performed by raising the temperature from 65 to 95 °C in 0.5 °C steps for 5 s each. To enable the quantification of PCR products, standard curves based on threshold cycles were produced by re-amplifying tenfold dilution series of PCR products from genomic DNA. Aliquots of each dilution (equivalent to 1 × 10^2^–1 × 10^8^ LSU copies) in triplicate were used as templates in qPCR. Means and standard deviations were calculated for qPCR products.

#### 2.3.3. Statistical Analysis

The one-way analysis of variance (ANOVA) and the pairwise multiple comparison procedures (Tukey’s HSD post-hoc test) were performed on log-transformed data using RStudio software [34,35].

### 2.4. Fungal Melanin Assay

#### 2.4.1. Pigment Extraction and Quantification

Melanin was extracted from the previous EVT2 and POS CTR samples following the optimized protocol for black fungi [36]. After the extraction, serial dilutions of purified pigments (1:5, 1:10, 1:20, 1:50 to 1:100) were made, allowing dissolution in an aqueous substrate (NaOH 1 M). To define the correlation between absorbance and wavelength, spectral measurements of all sample solutions were recorded via UV-Vis spectrophotometry as described in Pacelli et al. [36].

To determine the concentration of extracted melanin samples, synthetic DHN (1,8-diydroxynaphthalene) melanin (Sigma-Aldrich) was prepared in NaOH 1 M at a concentration of 500 mg/mL, and a standard curve at 650 nm (A_650_) was obtained as reported in Raman and Ramasamy [37]. Dilutions at 1:5 for the OS and S-MRS samples and at 1:50 for the P-MRS samples were selected for measuring the A_650_.

#### 2.4.2. Pigment Detection

Raman spectroscopic analysis was performed directly on the previous EVT2 samples at the Raman Laboratory of the German Aerospace Center (DLR) in Berlin, with an alpha300 R Confocal Raman microscope (WITec) at room temperature and under ambient atmospheric conditions, according to the optimized protocol by Böttger et al. [29].

The Raman laser excitation wavelength was 532 nm, and the spectral resolution was 4–5 cm^−1^. A Nikon 10× objective with a 0.25 numerical aperture was used to focus the laser on a 1.5 μm spot. Before the analysis, spectral calibration was performed with pure silicon and paracetamol test samples. For spectral data acquisitions, image scans were collected following the protocol reported by Pacelli et al. [38] and the spectral data evaluation and processing—including background evaluation—were performed with WITec Project FOUR software.

### 2.5. Fungal Organic Compound Detection

FT-IR spectroscopic analysis was performed directly on the previous EVT2 samples at the Planetary Emissivity Laboratory of DLR in Berlin.

Spectra were recorded in triplicate on the Vertex 80v FT-IR Spectrometer (Bruker) in reflectance mode and read at a resolution of 4 cm^−1^ in the wavenumber region of 400–10,000 cm^−1^ with Spectragryph software [39]. Measurements have been performed under evacuated atmosphere to remove atmospheric features from the spectra.

For analyzing the presence of specific organic molecules, the region of interest, 600–4000 cm^−1^, was cropped.

## 3. Results

### 3.1. DNA Damage Assessment

The integrity of genomic DNA samples was tested by assessing its ability to serve as a PCR template after the polychromatic UV irradiation treatment.

**Figure 1:**
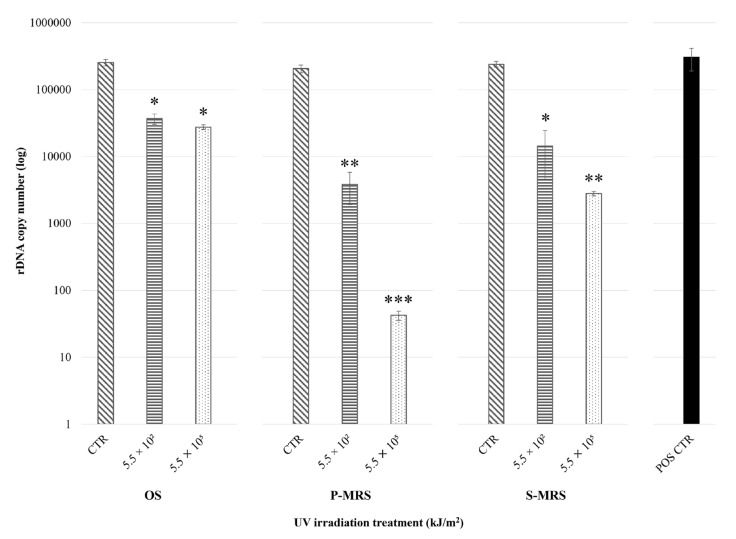
Real-time qPCR of an LSU gene fragment (~330 bp) from *C. antarcticus* after irradiation treatment within EVT2 simulations. On the x-axis, increasing polychromatic UV irradiation doses (CTR: non-irradiated sample; 5.5 × 10^2^ kJ/m^2^; 5.5 × 10^5^ kJ/m^2^: irradiated samples) for each substrate (OS: Antarctic sandstone; P-MRS: Phyllosilicatic Mars Regolith Simulant; S-MRS: Sulfatic Mars Regolith Simulant); on the y-axis, the number of amplified target gene copies (in logarithmic scale). Irradiation treatments were compared within analogues, whereas the positive control (POS CTR: *C. antarcticus* grown on MEA under physiological conditions) is shown for reference purposes. Statistically significant difference within substrate samples was calculated with Tukey’s test (* < 0.05; ** < 0.001; *** < 0.0001); no significant differences were reported between each CTR and the POS CTR (*p*-value < 0.05).

The results show reduced gene target amplifications when increasing the irradiation dose for all tested samples (Figure 1). Overall, an average of ~3·10^5^ copies were amplified in the POS CTR, whereas an average of ~2 × 10^5^ copies were amplified in the non-irradiated (i.e., CTR) samples, ~1.7 × 10^4^ in the low-irradiated (i.e., 5.5 × 10^2^ kJ/m^2^) samples, and ~1·10^4^ in the high-irradiated (i.e., 5.5 × 10^5^ kJ/m^2^) samples. Particularly, both OS 5.5 × 10^2^ and OS 5.5 × 10^5^ showed significantly fewer amplified copies than OS CTR (14% and 11%, respectively), as well as P-MRS 5.5 × 10^2^ kJ/m^2^ and P-MRS 5.5 × 10^5^ kJ/m^2^ with P-MRS CTR (2% and 0.02%, respectively), and S-MRS 5.5 × 10^2^ kJ/m^2^ and S-MRS 5.5 × 10^5^ kJ/m^2^ with S-MRS CTR (6% and 1%, respectively).

To summarize, the amplification was successful in all OS, P-MRS, and S-MRS samples, even in the high-irradiated P-MRS (5.5 × 10^5^ kJ/m^2^) sample, which reported fewer than 100 copies.

### 3.2. Pigment Characterization by UV-Vis Spectrophotometry

**Figure 2:**
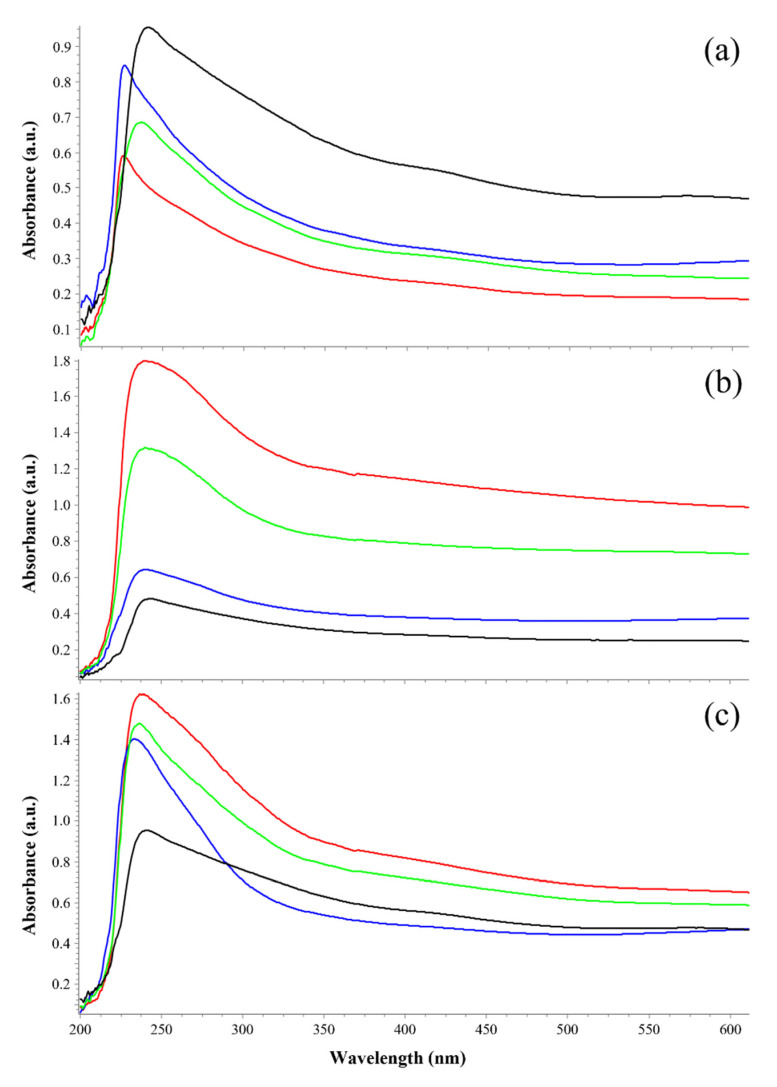
Ultraviolet–visible (UV-Vis) spectra of extracted melanin pigments from *C. antarcticus* dried colonies mixed with (**a**) OS: Antarctic sandstone; (**b**) P-MRS: Phyllosilicatic Mars Regolith Simulant; (**c**) S-MRS: Sulfatic Mars Regolith Simulant, after exposure to increasing polychromatic UV irradiation doses (CTR: red line; 5.5 × 10^2^ kJ/m^2^: blue line; 5.5 × 10^5^ kJ/m^2^: green line). On the x-axis, increasing wavelengths (in nm); on the y-axis, absorbance values (in absorbance units). Spectral profiles were compared within analogues, while the positive control (POS CTR: *C. antarcticus* grown on MEA under physiological conditions) is shown as the melanin spectrum reference (black line).

Due to its dark color, only melanin among all the biological pigments absorbs all visible wavelengths [40]. The nature of the purified pigments was thus confirmed by their spectral properties (Figure 2). Sample solutions were scanned from the UV to visible wavelength range (200 to 800 nm). Overall, every spectrum showed a strong absorbance in the UV region, reaching maximum values at ~230 nm and progressively decreasing as the wavelength increased. This property is due to the presence of a complex polymeric structure in the melanin molecule [41]. Particularly, as regards the POS CTR spectrum, all P-MRS and S-MRS spectra showed similar spectral profiles but with wider and narrower absorption peaks in the UV region, respectively.

**Figure 3:**
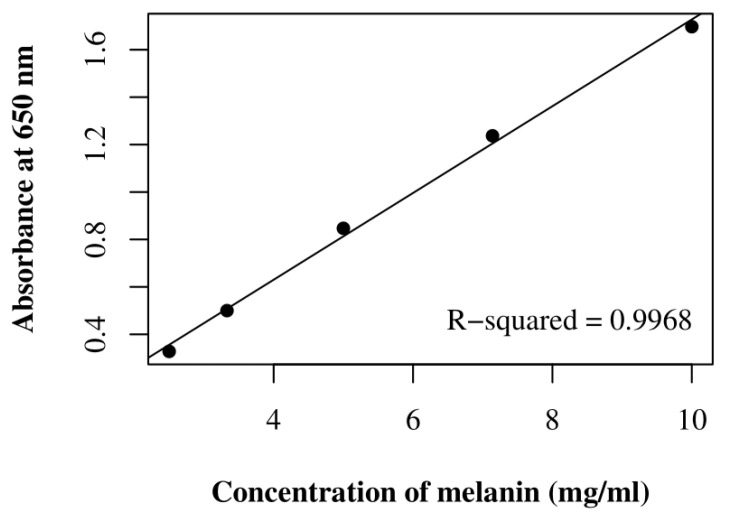
A_650_ standard curve of synthetic DHN melanin, which was calculated according to Raman and Ramasamy [37]. On the x-axis, the melanin concentration (in mg/mL); on the y-axis, the absorbance value at 650 nm (in absorbance units).

To quantify the sample of purified pigments, their concentration was estimated by comparing the absorbance values at 650 nm of each sample with the A_650_ standard curve of the synthetic DHN melanin (Figure 3). The mean and standard deviation values for melanin concentrations (in mg/mL) and the maximum absorbance wavelengths (in nm) are reported in Table 2.

### 3.3. Pigment Characterization by Raman Spectroscopy

**Figure 4:**
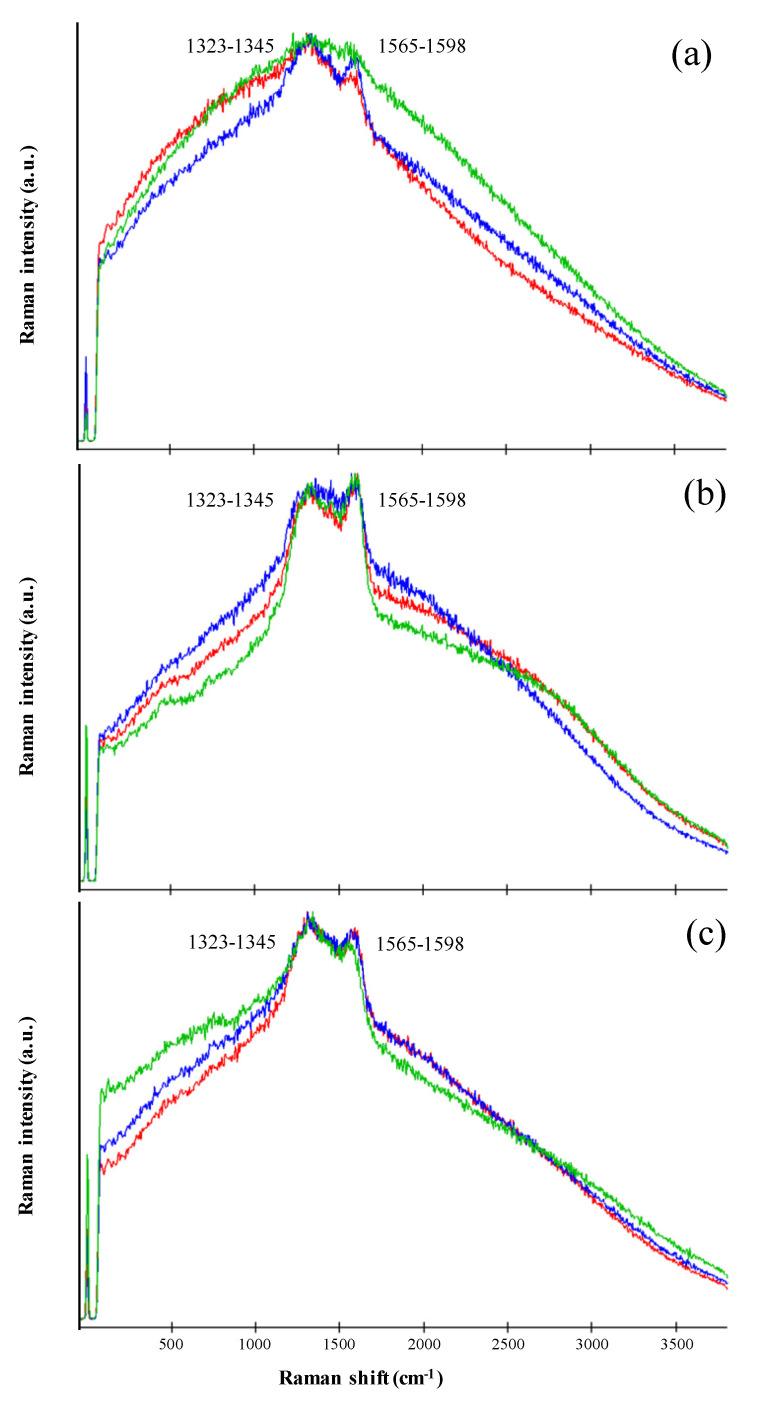
Raman spectra of *C. antarcticus* dried colonies mixed with (**a**) OS: Antarctic sandstone; (**b**) P-MRS: Phyllosilicatic Mars Regolith Simulant; (**c**) S-MRS: Sulfatic Mars Regolith Simulant, after exposure to polychromatic UV irradiation (CTR: red line; 5.5 × 10^2^ kJ/m^2^: blue line; 5.5 × 10^5^ kJ/m^2^: green line). On the x-axis, Raman shift (in cm^−1^); on the y-axis, Raman intensity of the scattered light (in arbitrary units). Shared peaks are highlighted within grey squares.

The Raman signal was obtained by focusing the exciting laser onto *C. antarcticus* dried colonies of the EVT2 samples without any previous preparation. All the acquired Raman spectra showed two clearly distinguishable broad peaks, located at 1323–1345 cm^−1^ and at 1565–1598 cm^−1^ (Figure 4). The Raman spectra obtained share key features with those reported in the literature for melanin pigments [38,42,43].

However, the definite assignment of the two melanin bands is not entirely agreed upon. Some authors ascribe both bands to the C–C stretching bonds within the aromatic rings, whereas others mark a contribution of aromatic C–N and C–H deformation bonds in the 1400 cm^–1^ region [44,45,46,47,48,49,50]. According to Culka et al. [43], this uncertainty is probably due to the vibrations of the constituent monomers, resulting in many overlapping Raman bands.

The specific positions of the main peaks serve as a proof that the samples did not undergo thermal degradation, and the signal acquired is of melanin and not burnt organic matter (or amorphous carbon), according to Pacelli et al. [38]. Furthermore, the presence of the peak around 1425 cm^−1^ is a clear sign of the *C. antarcticus*’ melanin [38].

### 3.4. Organic Compounds Characterization by FT-IR Spectroscopy

FT-IR spectroscopy was used to confirm the melanic nature of the *C. antarcticus* pigment, and to verify the possible presence of further organic compounds after exposure to UV irradiation treatment. 

**Figure 5:**
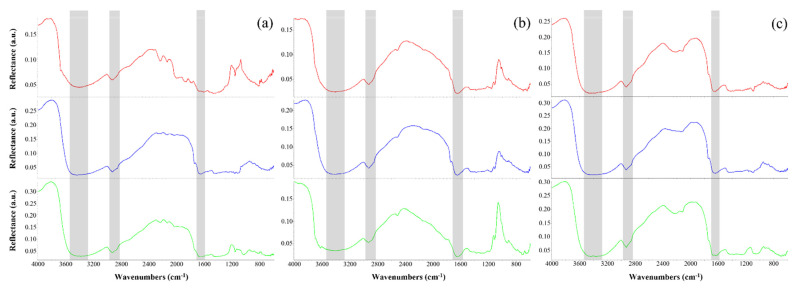
Fourier Transform Infrared (FT-IR) spectra of *C. antarcticus* dried colonies mixed with (**a**) OS: Antarctic sandstone (5.5 × 10^2^ kJ/m^2^: blue line; 5.5 × 10^5^ kJ/m^2^: green line), (**b**) P-MRS: Phyllosilicatic Mars Regolith Simulant (5.5 × 10^2^ kJ/m^2^: blue line; 5.5 × 10^5^ kJ/m^2^: green line) and (**c**): S-MRS: Sulfatic Mars Regolith Simulant (5.5 × 10^2^ kJ/m^2^: blue line; 5.5 × 10^5^ kJ/m^2^: green line). On the x-axis, decreasing wavenumbers (in cm^−1^); on the y-axis, reflectance values (in arbitrary units). Shared peaks are highlighted within grey squares.

Spectra were obtained directly from dried fungal colonies of the EVT2 samples without any previous preparation. All the acquired FT-IR spectra (Figure 5) revealed a strong, broad band at 3461–3372 cm^−1^, which was attributed to the O–H groups linked by the NH_2_ groups of phenolic and aromatic amino functions present in pyrrolic and indolic molecules [51,52,53]. Instead, the band duplet at 2931–2926 cm^−1^ and 2858–2851 cm^−1^ was ascribed to the CH stretching of saturated aliphatic hydrocarbons [46,54,55]. The small bands occurring between 1669 and 1632 cm^−1^ can be assigned to the alkenyl C=C and C=O stretching of the carboxylic or amide functions present in proteins [53,56,57,58]. The broad spectral region between 1250 and 1000 cm^−1^ can be related to phosphate groups, as well as the C–O, C–N, and C–O–C absorption peaks of carbohydrates or lipids [46,57,59,60]. Below 1000 cm^−1^, the absorption bands were usually weak, and mostly could be assigned to aromatic C–H and O–H out-of-plane deformations [53,57,58]. Other specific absorption bands have been identified in Table 3.

## 4. Discussion

In this work, we investigated the stability of biomolecules in the black fungus *C. antarcticus* after exposure to the ground-based simulations performed in preparation for the space-exposure BIOMEX project and in support of the biosignature detection method planned for the explorative mission on Mars.

The search for life on Mars is necessarily informed by terrestrial biomolecules, the only form of life that we know; furthermore, it is reasonable that life on Mars could be related to life on Earth due to the massive meteoritic exchange between Earth and Mars [64,65,66]. In addition, several papers have demonstrated that terrestrial life, if it landed on Mars, would be able to resist and live on Mars when protected by regolith from the harsh radiation environment of the Martian surface.

A prime consideration for the detection of organic biosignatures on Mars is their long-term preservation on the surface, where the presence of UV and ionizing radiation is one of the main damaging factors. Although the preservation of complex organic molecules in the shallow subsurface (~10 cm) of Martian rocks drops 1000-fold due to ionizing radiation [67], there are recent indications that the rapid burial of organic molecules, short surface exposures thanks to slower weathering rates, and the characteristics of certain mineral depositions are key factors enhancing biosignature preservation in the subsurface of Mars [68,69]. Therefore, we exposed dried fungal colonies, once grown on Martian rock analogues, to increasing doses of Mars-relevant polychromatic UV irradiation (200–400 nm) up to 5.5 × 10^5^ kJ/m^2^, which simulates 369.87 days on the Martian surface [30]. Here, we investigate the stability of the fungus itself and its related biomolecules in relation to the potential detectability of Martian biosignatures.

*C. antarcticus* DNA was showed to be resistant to the increasing UV doses, since its detection and amplification was achieved in all samples, even in those highly irradiated (5.5 × 10^5^ kJ/m^2^) and using very low DNA concentrations (0.018 ng/μL) (Figure 1). Although further data are required to confirm any potential role of the different substrates in providing protection against UV radiation, greater damages were reported for samples grown on Martian analogues. Although nucleic acids are organic compounds that are highly sensitive to degradation over geological timescales [1] and their preservation may be precluded [70], labile/polar compounds of these types are utile to assessing whether life processes are currently active, or were active in recent geological times [3,71]. Hence, we corroborate the possibility of using DNA as a potential biosignature in the search for extant or recently extinct life forms on Mars, considering its unambiguous biological origin. Since the Viking missions in the 1970s, nucleic acid detection instruments have not been included onboard life-detection rovers; however, the miniaturization of PCR devices could allow their integration in future missions. Indeed, the development of, e.g., the MinION device (Oxford Nanopore Technologies), a portable nanopore real-time sequencer that requires just small DNA or RNA amounts, would enable the clear detection of signs of life [72].

Among the other potential biosignatures investigated, we have focused on melanin pigments (present in all domains of terrestrial life) as they belong to those biomolecule classes (i.e., lipids and biopolymers) that exhibit the highest molecular stability and potential preservation among all organic compounds on Earth, i.e., over timescales of billions of years [2,3], while also being abundant in *C. antarcticus* cell walls [28] and well-known to be involved in photoprotection [43,73,74]. We used three different approaches to detect the pigments: (**i**) UV-Vis spectrophotometry; (**ii**) Raman spectroscopy; (**iii**) FT-IR spectroscopy.

(**i**) Since dark melanin pigments act as a blackbody, they absorb radiation at all visible wavelengths; the percentage of absorption is generally greater in the UV region and decreases progressively as the wavelength is increased to the far-red region [40]. The UV-Vis results of the purified pigments reported the typical spectral profile of fungal melanins [37,75,76,77] in the OS, P-MRS, and S-MRS spectra of all three irradiation treatments (Figure 2), showing the maximum absorbance peak at around 230 nm (Table 2). The greater absorption in the UV region is a property of aromatic organic compounds [78], and this absorbance peak suggests the detectability of melanin only: if proteins and nucleic acids were present, the peak would be shifted to the wavelength region between 250 and 320 nm [79].

Melanin has been proven to be well detectable and quantifiable (Table 2). Nevertheless, it has been noted that the P-MRS analogue, because it contains phyllosilicatic minerals or clays, presents high adsorption/absorption properties, with respect to the other substrates. It is likely that some minerals were still present in the purified melanin pigments interfering with the UV-Vis analysis, as reported in Pacelli et al. [74]; the presence of minerals may have altered melanin absorbance values at 650 nm, thus explaining the unusual spectral profile with a second large peak around 245 nm. In the context of searching for traces of extinct or extant life on Mars, the melanin concentration results from both P-MRS and S-MRS samples (Table 2) led us to assume that, although the estimation of melanin concentration from fungal colonies in Martian analogue regoliths can be altered by the presence of the soil itself, it is still possible to clearly detect the absorbance of purified melanin pigments, even with the presence of mineral compounds and in irradiated samples (up to 5.5 × 10^5^ kJ/m^2^).

(**ii**) The UV-Vis results of melanin pigment identification are supported by data from Raman and FT-IR analyses (Figure 4 and Figure 5). The sample spectra clearly show the presence of unaltered melanin pigments, even in samples exposed at a dose of 5.5 × 10^5^ kJ/m^2^. The specific peaks’ positions, and the presence of the intermediate peak at 1425 cm^−1^, allow us to discriminate melanin pigments from organic or inorganic carbon, which have similar features in the Raman spectrum, according to our previous study [38]. Neither the UV irradiation treatment nor dried conditions altered the melanin spectral properties, and thus melanin was successfully detected in all samples. Previous works have reported the degradative effect of UV and ionizing radiation on photosynthetic pigments (e.g., carotenoids), with the significant diminishment of spectral peak intensity and fluorescence [5,80,81]. Our results show the absence of the phenomenon on melanin pigments, demonstrating a successful detection even in samples irradiated with high UV doses (i.e., 5.5 × 10^5^ kJ/m^2^). This corroborates the initial proposal of using melanin as a potential biosignature due to its remarkable detectability in highly UV-irradiated environments.

(**iii**) Considering that two IR instruments are included in the ExoMars payloads [82,83,84,85], along with a Raman Laser Spectrometer, it is important to study biomolecules with a spectroscopic approach. FT-IR spectroscopy is an absorption-based optical analytical technique. The major benefits of using FT-IR imaging over traditional methods are that it is non-disruptive, label-free, and requires a very limited amount of samples. Through the acquisition of a biochemical fingerprint, FT-IR can provide information about the content, structure, and chemical modification of major biomolecules present in the investigated sample [86]. Raman and FT-IR spectroscopies are related techniques with respective complementary spectra [87]. The synergy of both techniques is a powerful tool when performing organic and inorganic compound characterization.

The results obtained by FT-IR spectroscopy reveal the presence of some main functional groups in all EVT2 spectra (Figure 5), and their presence was related to common organic compounds (Table 3). For instance, the ubiquitous presence of peaks at 3461–3372 cm^−1^, 2931–2926 cm^−1^, 2858–2851 cm^−1^, and 1669–1632 cm^−1^, along with the narrow one at 1061–1018 cm^−1^, is related to *C. antarcticus’* melanin pigments [36]. In particular, peaks at 3461–3372 cm^−1^ and 1669–1632 cm^−1^ are generally attributed to melanin pigments [53,88,89,90], while additional peaks at 2931–2926 cm^−1^, 2858–2851 cm^−1^ and 1061–1018 cm^−1^ are typically attributed to fungal melanins [37,76,91,92]. Besides this, the peaks observed in the fingerprint region of all spectra could eventually be identified as phenolic compounds, whose presence could be correlated to that of fungal melanins or other secondary metabolites [42,57].

As for the detection of fungal biomolecules other than melanin pigments, many narrow peaks between 1254 and 1018 cm^−1^ related to carbohydrates as well as to amino and phosphate compounds could be associated with polysaccharides of the fungal cell wall (e.g., chitosan, β-glucan, mannan, etc.) [46,59,60,93]. Furthermore, the peaks observed in relation to the amide I band (1669-1632 cm^−1^), the CH_2_ bending signal (1462–1445 cm^−1^), and CO stretching (1164–1158 cm^−1^) suggest the detection of chitin-characteristic vibrations [60,93,94,95]. To conclude, the FT-IR results confirm the presence of melanin pigments in all EVT2 samples; this evidence corroborates the suggestion of using the biomolecule as a biosignature, since it is highly detectable via different analytical techniques. Furthermore, all these results show the importance of using multiple and interconnected analytical techniques in the search for life, since they can provide complementary and useful information for detecting and characterizing biosignatures.

Moreover, our outcomes have identified chitin, the main component of the fungal cell wall, as a resistant biomolecule to UV irradiation. So far, it has also been reported in the context of the BIOMEX experiments via Raman analysis of *Circinaria gyrosa*’s fungal symbiont [96]. Chitin is known as one of the few biosignatures specific to fungi, and it has been reported in organically preserved fossils [97]. Nevertheless, the detection of chitin in geological materials is a difficult task: its occurrence in 25-million-year-old fossils has shown that the primary criterion for chitin preservation is not geological time, but rather the conditions of the depositional environment and the absence of diagenetic processes, such as alteration and decomposition [98]. Following the previous interpretation of FT-IR results, we consider the possibility of using chitin as a potential, stable biosignature, since it is detectable in fungal samples even after exposure to high UV irradiation (i.e., 5.5 × 10^5^ kJ/m^2^ dose) and is intrinsically related to terrestrial life.

## 5. Conclusions

In the light of previous investigations [36,43,73,74], we confirm the potential of using melanin pigments as a possible biosignature, and propose the further study of the chitin molecule as a possible new one, because both are highly stable biosynthesized molecules that could still be detected if exposed to extreme UV radiation—an extreme environmental condition that normally occurs on the Martian surface.

Even if the Martian subsurface offers protection against solar UV radiation and those oxidizing conditions found at the surface (only a few millimeters of dust coverage is required to shield against harmful UV radiation for survivable durations, in contrast to shorter-wavelength ionizing radiation that could penetrate up to 1.5 m beneath the surface) [99], this work addresses the case of recently surface-exposed rocks, where the photolytic destruction of organic compounds can rapidly occur. Therefore, this work can help broaden our knowledge on those terrestrial biomolecules that could be searched for in upcoming life-detection missions.

## Figures and Tables

**Table 1:** Sample exposure conditions during the UV irradiation treatment of the EXPOSE-R2 Experiment Verification Tests part 2 (EVT2) from the BIOMEX project.

Polychromatic UV Irradiance with SOL2000 (200–400 nm)	Irradiation Time/Sample	Resulting Fluence/Sample ^1^	Simulated Martian Days ^2^	Sample Set Substrate
0 J/(s·m^2^) → CTR (laboratory control)	0 s	0 kJ/m^2^	-	MEA + OS/ MEA + P-MRS/ MEA + S-MRS
0 J/(s·m^2^) → Dark (transport control)	0 s	0 kJ/m^2^	-	
1271 J/(s·m^2^)	7 min, 12 s	5.5 × 10^2^ kJ/m^2^ (0.1% ND ^3^)	0.37	
1271 J/(s·m^2^)	1 h, 12 min	5.5 × 10^3^ kJ/m^2^ (1.0% ND ^3^)	3.70	
1271 J/(s·m^2^)	30 h	1.4 × 10^5^ kJ/m^2^	94.15	
1271 J/(s·m^2^)	60 h	2.7 × 10^5^ kJ/m^2^	181.57	
1271 J/(s·m^2^)	120 h	5.5 × 10^5^ kJ/m^2^	369.87	
Physiological conditions → POS CTR (positive control)	0 s	0 kJ/m^2^	-	MEA

^1^ Each set of samples was irradiated with one of the applied fluences (from 5.5 × 10^2^ kJ/m^2^ up to 5.5 × 10^5^ kJ/m^2^) in the respective irradiation times, using a Solar Simulator with 1271 J/m^2^, as listed in the table. ^2^ Calculated from Cockell et al. [30]. ^3^ ND: neutral density filter. Modified from [26].

**Table 2:** Mean (± S.D.) values of the melanin concentration (in mg/mL) and the maximum UV absorbance wavelength (in nm) of purified pigments from the selected EVT2 and POS CTR samples.

Sample	Concentration (mg/mL)	Absorption Peak (nm)
OS CTR	5.271 (±2.705)	228 (±4)
OS 5.5 × 10^2^ kJ/m^2^	12.116 (±3.708)	230 (±6)
OS 5.5 × 10^5^ kJ/m^2^	6.758 (±7.760)	233 (±7)
P-MRS CTR	322.409 (±103.428)	234 (±9)
P-MRS 5.5 × 10^2^ kJ/m^2^	100.973 (±22.709)	236 (±6)
P-MRS 5.5 × 10^5^ kJ/m^2^	237.980 (±134.799)	234 (±9)
S-MRS CTR	24.005 (±15.427)	234 (±6)
S-MRS 5.5 × 10^2^ kJ/m^2^	19.811 (±3.545)	232 (±7)
S-MRS 5.5 × 10^5^ kJ/m^2^	22.096 (±14.595)	233 (±6)
POS CTR	20.136 (±5.752)	233 (±6)

OS: Antarctic sandstone; P-MRS: Phyllosilicatic Mars Regolith Simulant; S-MRS: Sulfatic Mars Regolith Simulant; CTR: non-irradiated sample; 5.5 × 10^2^ kJ/m^2^ and 5.5 × 10^5^ kJ/m^2^: irradiated samples; POS CTR: *C. antarcticus* grown on MEA under physiological conditions.

**Table 3:** Absorption bands (in cm^−1^) observed in the EVT2 FT-IR spectra and their possible molecular bonding interpretation.

Wavenumber (cm^−1^)	Molecular Bond Interpretation	References
3461–3372	NH_2_/OH stretching of phenols and aromatic amines in indolic and pyrrolic systems	[53]
2931–2926	CH_2_ asymmetric stretching of saturated aliphatic groups in fatty acids	[55,56]
2858–2851	CH_2_ symmetric stretching of saturated aliphatic groups in fatty acids	[55,56]
2515–2511	OH stretching in carboxylic acids	[58,61]
2249	Acetylenic C≡C/aliphatic nitrile C≡N stretching	[56,58]
2233–2211	Acetylenic C≡C/aromatic nitrile C≡N stretching	[56,58]
2136–2114	Acetylenic C≡C/aliphatic isonitrile N≡C stretching	[56,58]
2037–1788	Several vibrations from overtones and combinations of substituted benzene rings	[61]
1743–1738	C=O stretching of aliphatic esters in triglycerides	[58,62]
1669–1632	Alkenyl C=C/C=O/C=N stretching of amide I in proteins	[56,58]
1462–1445	CH_2_ scissoring/CH_3_ asymmetric bending of aliphatic compounds in proteins or fatty acids	[58,63]
1422–1415	OH in-plane bending in carboxylic acids	[61]
1380–1374	CH_3_ symmetric bending of aliphatic compounds in proteins or fatty acids	[58,63]
1254–1252	P=O asymmetric/CO stretching of aliphatic phosphorus compounds, aromatic ethers and esters in carbohydrates or lipids	[58,59]
1193–1190	PO/CN/CO stretching of aromatic amines, phosphates, and phenols in carbohydrates or lipids	[58,61]
1164–1158	CO stretching of aliphatic ethers, esters, and alcohols in carbohydrates	[58,61]
1122–1085	CN/COC asymmetric/CO stretching of aliphatic amines, ethers, esters, and alcohols in carbohydrates	[57,61]
1061–1018	POC asymmetric/CO stretching of aliphatic phosphorus compounds, ethers, and alcohols in carbohydrates	[57,58]
928–921	COH out-of-plane bending in carboxylic acids	[58]
901–814	CH out-of-plane bending of benzenes	[57,61]
721–712	–(CH_2_)_n_– rocking of methylene chains in hydrocarbons	[58,63]
700–687	Olefinic *cis*-CH/phenolic OH out-of-plane bending	[61]
675–642	Acetylenic C=CH bending	[56]

## Data Availability

The data presented in this study are available within the manuscript.

## References

[1] Aerts J.W., Röling W.F., Elsaesser A., Ehrenfreund P. (2014). Biota and biomolecules in extreme environments on Earth: Implications for life detection on Mars. Life.

[2] Engel M.H., Macko S.A. (1993). Organic Geochemistry: Principles and Applications.

[3] Simoneit B.R., Summons R.E., Jahnke L.L. (1998). Biomarkers as tracers for life on early Earth and Mars. Orig. Life Evol. Biosph..

[4] Cockell C.S., Schuerger A.C., Billi D., Friedmann E.I., Panitz C. (2005). Effects of a simulated martian UV flux on the cyanobacterium, Chroococcidiopsis sp.029. Astrobiology.

[5] Dartnell L.R., Patel M.R., Storrie-Lombardi M.C., Ward J.M., Muller J.P. (2012). Experimental determination of photostability and fluorescence-based detection of PAHs on the Martian surface. Meteorit. Planet. Sci..

[6] Dartnell L.R., Patel M.R. (2014). Degradation of microbial fluorescence biosignatures by solar ultraviolet radiation on Mars. Int. J. Astrobiol..

[7] Poch O., Noblet A., Stalport F., Correia J.J., Grand N., Szopa C., Coll P. (2013). Chemical evolution of organic molecules under Mars-like UV radiation conditions simulated in the laboratory with the “Mars organic molecule irradiation and evolution” (MOMIE) setup. Planet. Space Sci..

[8] Friedmann E.I., Ocampo-Friedmann R. (1984). The Antarctic cryptoendolithic ecosystem: Relevance to exobiology. Orig. Life.

[9] Patel M.R., Zarnecki J.C., Catling D.C. (2002). Ultraviolet radiation on the surface of Mars and the Beagle 2 UV sensor. Planet. Space Sci..

[10] Rothschild L.J., Cockell C.S. (1999). Radiation: Microbial evolution, ecology, and relevance to Mars missions. Mutat. Res. Fundam. Mol. Mech. Mutagen..

[11] Gómez-Elvira J., Armiens C., Carrasco I., Genzer M., Gómez F., Haberle R., Hamilton V.E., Harri A.-M., Kahanpää H., Kemppinen O. (2014). Curiosity’s rover environmental monitoring station: Overview of the first 100 sols. J. Geophys. Res. Planets.

[12] Kuhn W.R., Atreya S.K. (1979). Solar radiation incident on the Martian surface. J. Mol. Evol..

[13] Schuerger A.C., Mancinelli R.L., Kern R.G., Rothschild L.J., McKay C.P. (2003). Survival of endospores of Bacillus subtilis on spacecraft surfaces under simulated Martian environments: Implications for the forward contamination of Mars. Icarus.

[14] Stalport F., Coll P., Szopa C., Cottin H., Raulin F. (2009). Investigating the photostability of carboxylic acids exposed to Mars surface ultraviolet radiation conditions. Astrobiology.

[15] Carrier B.L., Kounaves S.P. (2015). The origins of perchlorate in the Martian soil. Geophys. Res. Lett..

[16] Encrenaz T., Greathouse T.K., Lefèvre F., Atreya S.K. (2012). Hydrogen peroxide on Mars: Observations, interpretation and future plans. Planet. Space Sci..

[17] Glavin D.P., Freissinet C., Miller K.E., Eigenbrode J.L., Brunner A.E., Buch A., Sutter B., Archer P.D., Atreya S.K., Brinckerhoff W.B. (2013). Evidence for perchlorates and the origin of chlorinated hydrocarbons detected by SAM at the Rocknest aeolian deposit in Gale Crater. J. Geophys. Res. Planets.

[18] Ming D.W., Archer P.D., Glavin D.P., Eigenbrode J.L., Franz H.B., Sutter B., Brunner A.E., Stern J.C., Freissinet C., McAdam A.C. (2014). Volatile and organic compositions of sedimentary rocks in Yellowknife Bay, Gale Crater, Mars. Science.

[19] Quinn R.C., Martucci H.F., Miller S.R., Bryson C.E., Grunthaner F.J., Grunthaner P.J. (2013). Perchlorate radiolysis on Mars and the origin of martian soil reactivity. Astrobiology.

[20] Hassler D.M., Zeitlin C., Wimmer-Schweingruber R.F., Ehresmann B., Rafkin S., Eigenbrode J.L., Brinza D.E., Weigle G., Böttcher S., Böhm E. (2014). Mars’ Surface Radiation Environment Measured with the Mars Science Laboratory’s Curiosity Rover. Science.

[21] Moores J.E., Smith P.H., Tanner R., Schuerger A.C., Venkateswaran K.J. (2007). The shielding effect of small-scale Martian surface geometry on ultraviolet flux. Icarus.

[22] Patel M.R., Bérces A., Kolb C., Lammer H., Rettberg P., Zarnecki J.C., Selsis F. (2003). Seasonal and diurnal variations in Martian surface ultraviolet irradiation: Biological and chemical implications for the Martian regolith. Int. J. Astrobiol..

[23] Schuerger A.C., Moores J.E., Clausen C.A., Barlow N.G., Britt D.T. (2012). Methane from UV-irradiated carbonaceous chondrites under simulated Martian conditions. J. Geophys. Res. Planets.

[24] Lopez-Reyes G., Pilorget C., Moral A.G., Manrique J.A., Sanz A., Berrocal A., Veneranda M., Rull F., Medina J., Hamm V. (2020). Raman Laser Spectrometer (RLS) calibration target design to allow onboard combined science between the RLS and MicrOmega instruments on the ExoMars rover. J. Raman Spectrosc..

[25] Kinch K.M., Madsen M.B., Bell J.F., Maki J.N., Bailey Z.J., Hayes A.G., Jensen O.B., Merusi M., Bernt M.H., Sørensen A.N. (2020). Radiometric calibration targets for the Mastcam-Z Camera on the Mars 2020 Rover mission. Space Sci. Rev..

[26] De Vera J.-P., Alawi M., Backhaus T., Baqué M., Billi D., Böttger U., Berger T., Bohmeier M., Cockell C.S., Demets R. (2019). Limits of life and the habitability of Mars: The ESA space experiment BIOMEX on the ISS. Astrobiology.

[27] Rabbow E., Rettberg P., Parpart A., Panitz C., Schulte W., Molter F., Schulte W., Jaramillo E., Demets R., Weiß P. (2017). EXPOSE R2: The astrobiological ESA mission on board of the International Space Station. Front. Microbiol..

[28] Selbmann L., de Hoog G.S., Mazzaglia A., Friedmann E.I., Onofri S. (2005). Fungi at the edge of life: Cryptoendolithic black fungi from Antarctic desert. Stud. Mycol..

[29] Böttger U., de Vera J.-P., Fritz J., Weber I., Hübers H.W., Schulze-Makuch D. (2012). Optimizing the detection of carotene in cyanobacteria in a Martian regolith analogue with a Raman spectrometer for the ExoMars mission. Planet. Space Sci..

[30] Cockell C.S., Catling D.C., Davis W.L., Snook K., Kepner R.L., Lee P., McKay C.P. (2000). The ultraviolet environment of Mars: Biological implications past, present, and future. Icarus.

[31] Vilgalys R., Hester M. (1990). Rapid genetic identification and mapping of enzymatically amplified ribosomal DNA from several Cryptococcus species. J. Bacteriol..

[32] Hopple J.S., Vilgalys R. (1999). Phylogenetic relationships in the mushroom genus Coprinus and dark-spored allies based on sequence data from the nuclear gene coding for the large ribosomal subunit RNA: Divergent domains, outgroups, and monophyly. Mol. Phylogenet. Evol..

[33] Selbmann L., Isola D., Zucconi L., Onofri S. (2011). Resistance to UV-B induced DNA damage in extreme-tolerant cryptoendolithic Antarctic fungi: Detection by PCR assays. Fungal Biol..

[34] R Core Team (2018). R: A Language and Environment for Statistical Computing.

[35] RStudio Team (2016). RStudio: Integrated Development for R.

[36] Pacelli C., Cassaro A., Maturilli A., Timperio A.M., Gevi F., Cavalazzi B., Stefan M., Ghica D., Onofri S. (2020). Multidisciplinary characterization of melanin pigments from the black fungus Cryomyces antarcticus. Appl. Microbiol. Biotechnol..

[37] Raman N.M., Ramasamy S. (2017). Genetic validation and spectroscopic detailing of DHN-melanin extracted from an environmental fungus. Biochem. Biophys. Rep..

[38] Pacelli C., Cassaro A., Baqué M., Selbmann L., Zucconi L., Maturilli A., Onofri S. (2021). Fungal biomarkers are detectable in Martian rock-analogues after space exposure: Implications for the search of life on Mars. Int. J. Astrobiol..

[39] Menges F. (2019). Spectragryph—Optical Spectroscopy Software. http://www.effemm2.de/spectragryph/.

[40] Bell A.A., Wheeler M.H. (1986). Biosynthesis and functions of fungal melanins. Annu. Rev. Phytopathol..

[41] Cockell C.S., Knowland J. (1999). Ultraviolet radiation screening compounds. Biol. Rev..

[42] Centeno S.A., Shamir J. (2008). Surface enhanced Raman scattering (SERS) and FTIR characterization of the sepia melanin pigment used in works of art. J. Mol. Struct..

[43] Culka A., Jehlička J., Ascaso C., Artieda O., Casero C.M., Wierzchos J. (2017). Raman microspectrometric study of pigments in melanized fungi from the hyperarid Atacama desert gypsum crust. J. Raman Spectrosc..

[44] De Gussem K., Vandenabeele P., Verbeken A., Moens L. (2005). Raman spectroscopic study of Lactarius spores (Russulales, Fungi). Spectrochim. Acta A Mol. Biomol. Spectrosc..

[45] Huang Z., Lui H., Chen M.X., Alajlan A., McLean D.I., Zeng H. (2004). Raman spectroscopy of in vivo cutaneous melanin. J. Biomed. Opt..

[46] Maquelin K., Choo-Smith L.P., Kirschner C., Ngo-Thi N.A., Naumann D., Puppels G.J. (2002). Vibrational spectroscopic studies of microorganisms. Handb. Vib. Spectrosc..

[47] Movasaghi Z., Rehman S., Rehman I.U. (2007). Raman spectroscopy of biological tissues. Appl. Spectrosc. Rev..

[48] Rösch P., Harz M., Peschke K.D., Ronneberger O., Burkhardt H., Popp J. (2006). Identification of single eukaryotic cells with micro-Raman spectroscopy. Biopolymers.

[49] Samokhvalov A., Liu Y., Simon J.D. (2004). Characterization of the Fe (III)-binding Site in Sepia Eumelanin by Resonance Raman Confocal Microspectroscopy. Photochem. Photobiol..

[50] Saif F.A., Yaseen S.A., Alameen A.S., Mane S.B., Undre P.B. (2020). Identification and characterization of Aspergillus species of fruit rot fungi using microscopy, FT-IR, Raman and UV–Vis spectroscopy. Spectrochim. Acta A Mol. Biomol. Spectrosc..

[51] Hou R., Liu X., Xiang K., Chen L., Wu X., Lin W., Zheng M., Fu J. (2019). Characterization of the physicochemical properties and extraction optimization of natural melanin from Inonotus hispidus mushroom. Food Chem..

[52] Jones R.A. (1970). Physicochemical Properties of Pyrroles. Adv. Heterocycl. Chem..

[53] Magarelli M., Passamonti P., Renieri C. (2010). Purification, characterization and analysis of sepia melanin from commercial sepia ink (Sepia Officinalis). Rev. CES Med. Vet. Zootec..

[54] Movasaghi Z., Rehman S., Rehman D.I. (2008). Fourier transform infrared (FTIR) spectroscopy of biological tissues. Appl. Spectrosc. Rev..

[55] Safar M., Bertrand D., Robert P., Devaux M.F., Genot C. (1994). Characterization of edible oils, butters and margarines by Fourier transform infrared spectroscopy with attenuated total reflectance. J. Am. Oil Chem. Soc..

[56] Coates J., Meyers R.A. (2000). Interpretation of infrared spectra, a practical approach. Encyclopedia of Analytical Chemistry: Applications, Theory and Instrumentation.

[57] Schulz H., Baranska M. (2007). Identification and quantification of valuable plant substances by IR and Raman spectroscopy. Vib. Spectrosc..

[58] Stuart B.H. (2004). Infrared Spectroscopy: Fundamentals and Applications.

[59] Kosa G., Kohler A., Tafintseva V., Zimmermann B., Forfang K., Afseth N.K., Tzimorotas D., Vuoristo K.S., Horn S.J., Mounier J. (2017). Microtiter plate cultivation of oleaginous fungi and monitoring of lipogenesis by high-throughput FTIR spectroscopy. Microb. Cell Fact..

[60] Salman A., Shufan E., Tsror L., Moreh R., Mordechai S., Huleihel M. (2014). Classification of Colletotrichum coccodes isolates into vegetative compatibility groups using infrared attenuated total reflectance spectroscopy and multivariate analysis. Methods.

[61] Shurvell H.F., Chalmers J.M., Griffiths P.R. (2006). Spectra–structure correlations in the mid-and far-infrared. Handbook of Vibrational Spectroscopy.

[62] Guillén M.D., Cabo N. (1999). Usefulness of the frequency data of the Fourier transform infrared spectra to evaluate the degree of oxidation of edible oils. J. Agric. Food Chem..

[63] Irudayaraj J., Sivakesava S., Kamath S., Yang H. (2001). Monitoring chemical changes in some foods using Fourier transform photoacoustic spectroscopy. J. Food Sci..

[64] Gladman B.J., Burns J.A., Duncan M., Lee P., Levison H.F. (1996). The exchange of impact ejecta between terrestrial planets. Science.

[65] Melosh H.J. (1988). The rocky road to panspermia. Nature.

[66] Mileikowsky C., Cucinotta F.A., Wilson J.W., Gladman B., Horneck G., Lindegren L., Melosh J., Rickman H., Valtonen M., Zheng J.Q. (2000). Natural transfer of viable microbes in space: 1. From Mars to Earth and Earth to Mars. Icarus.

[67] Pavlov A.A., Vasilyev G., Ostryakov V.M., Pavlov A.K., Mahaffy P. (2012). Degradation of the organic molecules in the shallow subsurface of Mars due to irradiation by cosmic rays. Geophys. Res. Lett..

[68] Hays L.E., Graham H.V., Des Marais D.J., Hausrath E.M., Horgan B., McCollom T.M., Parenteau M.N., Potter-McIntyre S.L., Williams A.J., Lynch K.L. (2017). Biosignature preservation and detection in Mars analog environments. Astrobiology.

[69] Williams A.J., Craft K.L., Millan M., Johnson S.S., Knudson C.A., Juarez Rivera M., McAdam A.C., Tobler D., Skok J.R. (2021). Fatty Acid Preservation in Modern and Relict Hot-Spring Deposits in Iceland, with Implications for Organics Detection on Mars. Astrobiology.

[70] National Research Council (2007). An Astrobiology Strategy for the Exploration of Mars.

[71] Summons R.E., Amend J.P., Bish D., Buick R., Cody G.D., Des Marais D.J., Dromart G., Eigenbrode J.L., Knoll A.H., Sumner D.Y. (2011). Preservation of Martian organic and environmental records: Final report of the Mars Biosignature Working Group. Astrobiology.

[72] Maggiori C., Stromberg J., Blanco Y., Goordial J., Cloutis E., García-Villadangos M., Parro V., Whyte L. (2020). The Limits, Capabilities, and Potential for Life Detection with MinION Sequencing in a Paleochannel Mars Analog. Astrobiology.

[73] Pacelli C., Bryan R.A., Onofri S., Selbmann L., Shuryak I., Dadachova E. (2017). Melanin is effective in protecting fast and slow growing fungi from various types of ionizing radiation. Environ. Microbiol..

[74] Pacelli C., Cassaro A., Aureli L., Moeller R., Fujimori A., Onofri S. (2020). The Responses of the Black Fungus Cryomyces antarcticus to High Doses of Accelerated Helium Ions Radiation within Martian Regolith Simulants and Their Relevance for Mars. Life.

[75] Meeßen J., Sánchez F.J., Sadowsky A., de la Torre R., Ott S., de Vera J.P. (2013). Extremotolerance and Resistance of Lichens: Comparative Studies on Five Species Used in Astrobiological Research II. Secondary Lichen Compounds. Orig. Life Evol. Biosph..

[76] Pal A.K., Gajjar D.U., Vasavada A.R. (2013). DOPA and DHN pathway orchestrate melanin synthesis in Aspergillus species. Med. Mycol..

[77] Suryanarayanan T.S., Ravishankar J.P., Venkatesan G., Murali T.S. (2004). Characterization of the melanin pigment of a cosmopolitan fungal endophyte. Mycol. Res..

[78] Yuan W., Burleigh S.H., Dawson J.O. (2007). Melanin biosynthesis by Frankia strain CeI5. Physiol. Plant..

[79] Mach H., Volkin D.B., Burke C.J., Middaugh C.R., Shirley B.A. (1995). Ultraviolet Absorption Spectroscopy. Protein Stability and Folding. Methods in Molecular Biology.

[80] Baqué M., Hanke F., Böttger U., Leya T., Moeller R., de Vera J.-P. (2018). Protection of cyanobacterial carotenoids’ Raman signatures by Martian mineral analogues after high-dose gamma irradiation. J. Raman Spectrosc..

[81] Dartnell L.R., Storrie-Lombardi M.C., Mullineaux C.W., Ruban A.V., Wright G., Griffiths A.D., Muller J.P., Ward J.M. (2011). Degradation of cyanobacterial biosignatures by ionizing radiation. Astrobiology.

[82] Bibring J.P., Hamm V., Pilorget C., Vago J.L. (2017). The micrOmega investigation onboard ExoMars. Astrobiology.

[83] De Sanctis M.C., Altieri F., Ammannito E., Biondi D., De Angelis S., Meini M., Mondello G., Novi S., Paolinetti R., Soldani M. (2017). Ma_MISS on ExoMars: Mineralogical characterization of the Martian subsurface. Astrobiology.

[84] Rull F., Maurice S., Hutchinson I., Moral A., Perez C., Diaz C., Colombo M., Belenguer T., Lopez-Reyes G., Parot Y. (2017). The Raman laser spectrometer for the ExoMars rover mission to Mars. Astrobiology.

[85] Vago J.L., Westall F., Pasteur Instrument Teams, Landing Site Selection Working Group, Other Contributors (2017). Habitability on early Mars and the search for biosignatures with the ExoMars Rover. Astrobiology.

[86] Kendall C., Isabelle M., Bazant-Hegemark F., Hutchings J., Orr L., Babrah J., Baker R., Stone N. (2009). Vibrational spectroscopy: A clinical tool for cancer diagnostics. Analyst.

[87] McCreery R.L. (2001). Raman spectroscopy for chemical analysis. Meas. Sci. Technol..

[88] Bonner T.G., Duncan A. (1962). Infra-red spectra of some melanins. Nature.

[89] Kannan P., Ganjewala D. (2009). Preliminary characterization of melanin isolated from fruits and seeds of Nyctanthes arbor-tristis. J. Sci. Res..

[90] Sajjan S., Kulkarni G., Yaligara V., Kyoung L., Karegoudar T.B. (2010). Purification and physiochemical characterization of melanin pigment from Klebsiella sp. GSK. J. Microbiol. Biotechnol..

[91] Paim S., Linhares L.F., Mangrich A.S., Martin J.P. (1990). Characterization of fungal melanins and soil humic acids by chemical analysis and infrared spectroscopy. Biol. Fertil. Soils.

[92] Sun S., Zhang X., Sun S., Zhang L., Shan S., Zhu H. (2016). Production of natural melanin by Auricularia auricula and study on its molecular structure. Food Chem..

[93] Forfang K., Zimmermann B., Kosa G., Kohler A., Shapaval V. (2017). FTIR spectroscopy for evaluation and monitoring of lipid extraction efficiency for oleaginous fungi. PLoS ONE.

[94] Ivshin V.P., Artamonova S.D., Ivshina T.N., Sharnina F.F. (2007). Methods for isolation of chitin-glucan complexes from higher fungi native biomass. Polym. Sci. Ser. B.

[95] Rinaudo M. (2006). Chitin and chitosan: Properties and applications. Prog. Polym. Sci..

[96] Böttger U., Meeßen J., Martínez-Frías J., Hübers H.W., Rull F., Sánchez F.J., de la Torre R., de Vera J.P. (2014). Raman spectroscopic analysis of the calcium oxalate producing extremotolerant lichen Circinaria gyrosa. Int. J. Astrobiol..

[97] Briggs D.E., Summons R.E. (2014). Ancient biomolecules: Their origins, fossilization, and role in revealing the history of life. BioEssays.

[98] Ivarsson M., Broman C., Holmström S.J., Ahlbom M., Lindblom S., Holm N.G. (2011). Putative fossilized fungi from the lithified volcaniclastic apron of Gran Canaria, Spain. Astrobiology.

[99] Cockell C.S., Raven J.A. (2004). Zones of photosynthetic potential on Mars and the early Earth. Icarus.

